# Diagnostic Accuracy of Spectral Doppler Indices and Sonoelastography in Predicting Malignancy in Breast Imaging Reporting and Database System 3 Breast Lesions With Histopathology as the Reference Standard

**DOI:** 10.7759/cureus.73481

**Published:** 2024-11-11

**Authors:** Linnet Prabakaran, Senthil Kumar Aiyappan, Sabari Ramesh, Ragitha Ramesh, Subhalakshmi Kumaran

**Affiliations:** 1 Radiodiagnosis, Sri Ramaswamy Memorial Medical College Hospital and Research Centre, Chengalpattu, IND

**Keywords:** bi-rads 3, b mode ultrasound, breast cancer, doppler pi and ri, strain elastography

## Abstract

Introduction

Breast cancer is a significant health concern in India, representing a large portion of all cancers affecting women and ranking as one of the most common cancers overall. Reliable diagnostic tools are essential for accurately predicting malignancy and reducing the need for unnecessary biopsies. A Breast Imaging Reporting and Database System (BI-RADS) 3 designation suggests a low likelihood of cancer, indicating that findings are likely benign. For these cases, short-term follow-up imaging is generally preferred over immediate biopsy as the probability of malignancy is minimal. This study evaluates the effectiveness of strain elastography, specifically the strain ratio, in predicting malignancy in BI-RADS 3 breast lesions in a cohort of 50 patients. Additionally, it examines the role of Doppler indices, including the Pulsatility Index (PI) and Resistance Index (RI). Histopathological analysis was used as the reference standard.

Methods

A descriptive cross-sectional study was conducted on 50 patients presenting with palpable breast lumps or abnormalities detected via ultrasonography or mammography. Conventional B-mode ultrasound examinations were performed on all the patients, and those with BI-RADS 3 lesions were identified. The Doppler technique was employed to calculate PI and RI values, followed by strain elastography to determine the strain ratio. Histopathological confirmation was performed for all patients.

Results

Histopathological analysis revealed that 92% (N=46) of the lesions were benign, while 8% (N=4) were malignant. For strain elastography, the sensitivity was 75%, specificity was 97.83%, positive predictive value (PPV) was 75%, negative predictive value (NPV) was 97.83%, and the diagnostic accuracy was 96%. For Doppler PI, the sensitivity was 75%, specificity was 95.65%, PPV was 60%, NPV was 97.78%, and the overall diagnostic accuracy was 94%. Similarly, for Doppler RI, the sensitivity was 75%, specificity was 95.65%, PPV was 60%, NPV was 97.78%, and the overall diagnostic accuracy was 94%.

Conclusion

B-mode ultrasound remains the first-line imaging investigation for evaluating breast masses. In BI-RADS 3 lesions, where the likelihood of malignancy is minimal, the combined use of strain elastography and Doppler PI and RI indices can serve as a valuable adjunct in predicting malignancy and reducing the need for unnecessary biopsies. Moreover, strain elastography demonstrates higher diagnostic accuracy compared to Doppler PI and RI.

## Introduction

Breast cancer is one of the most prevalent cancers in India, accounting for approximately 28% of all female cancers and ranking as the second most common cancer overall [1]. This statistic highlights the urgency of early diagnosis and screening for breast carcinoma. Various imaging modalities used to evaluate breast masses include greyscale ultrasonography, Doppler ultrasound, strain and shear wave elastography, mammography, and magnetic resonance imaging (MRI) [2]. Tumors, being metabolically hyperactive, require an increased supply of oxygen and nutrients, which drives the formation of new blood vessels, a process known as angiogenesis [3]. Doppler ultrasound, through its color and spectral techniques, can detect neovascularization, a key marker of malignancy, and thus help distinguish between benign and malignant tumors [4]. Strain elastography measures tissue stiffness by evaluating tissue deformation in response to an external force. Generally, malignant tissues are stiffer than benign ones [5]. Combining Doppler ultrasound with elastography has been shown to improve diagnostic accuracy to 88.2% from 72.6% with routine B-mode ultrasound [6].

Given the 2% likelihood of malignancy in a Breast Imaging Reporting and Data System (BI-RADS) 3 lesion, it is crucial to utilize reliable diagnostic tools that can predict malignancy and reduce the need for unnecessary biopsies [7]. Despite their potential, spectral Doppler parameters are not yet universally adopted in routine breast ultrasound examinations at many imaging centers. Ultrasound is an important adjunct to digital mammography, particularly in young women at high risk of breast cancer and those with dense breast tissue, as it is a non-invasive and radiation-free modality [8]. Integrating spectral Doppler ultrasound and strain elastography into routine practice could help reduce unnecessary biopsies and inform treatment planning strategies [9]. Both spectral Doppler parameters and strain elastography hold significant promise for the future of breast cancer diagnostics [10]. This study aims to assess the accuracy of strain elastography and Doppler indices, including the Pulsatility Index (PI) and Resistance Index (RI), in predicting malignancy in BI-RADS 3 lesions.

## Materials and methods

**Figure 1:**
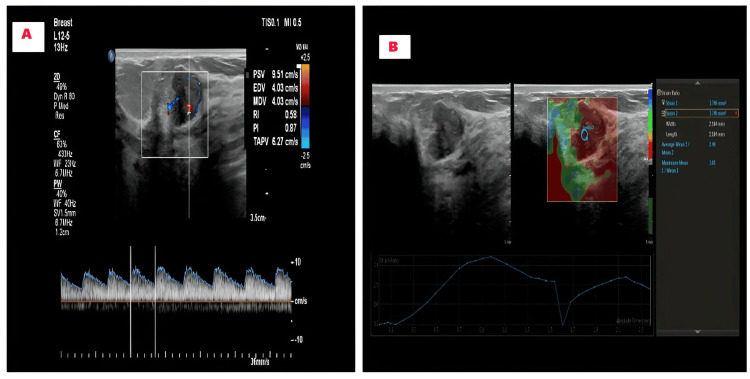
Assessing BI-RADS 3 lesions using spectral Doppler and strain elastography A: Spectral Doppler measured the PI and RI B: The strain ratio was calculated using strain elastography technique BI-RADS 3: Breast imaging reporting and data system; PI: pulsatility index; RI: resistance index.

This study was conducted in the Department of Radiodiagnosis at SRM Medical College Hospital and Research Centre, Kattankulathur, Chengalpattu, for one year and six months (September 2022 to March 2024). Patients aged 15-70 years presenting with breast lumps underwent routine grayscale ultrasonography and BI-RADS 3 lesions were identified. The inclusion criteria consisted of BI-RADS 3 lesions that exhibited at least one vascular signal on Doppler ultrasonography (USG). Exclusion criteria included patients with breast lesions other than BI-RADS 3, small lesions for which biopsy was not feasible, and patients who did not consent to histopathological examination (HPE) or fine needle aspiration cytology (FNAC). Following ethical clearance from the SRM Institutional Ethics Committee (SRMIEC-ST0722-019) and the acquisition of written informed consent, patients were enrolled in the study. Initially, a conventional B-mode ultrasound examination was performed using a linear probe (5-12 MHz) on the Philips Affiniti 70 ultrasound machine (Koninklijke Philips N.V., Amsterdam, Netherlands), and BI-RADS 3 lesions were identified. Using color and spectral Doppler techniques, PI and RI values were calculated. Next, strain elastography was performed by placing a Region of Interest (ROI) box to include both normal and abnormal tissues. The relative stiffness of the tissue was represented in color, with areas ranging from blue (soft) to red (stiff), and the stiffest area, typically displayed in red, was selected for analysis. After applying adequate compression, the strain ratio was calculated by comparing the stiffest area to the adjacent softer normal breast parenchyma (Figures 1A, 1B).

Following the imaging studies, patients underwent FNAC or biopsy. The final results from the FNAC or biopsy were compared with the Doppler and elastography findings. Cut-off values for predicting malignancy in BI-RADS 3 lesions were set at PI>1, RI>0.8, and strain ratio>3 [10]. Descriptive statistics were used to summarize numerical and categorical variables, which were presented as frequencies and percentages. For inferential statistics, confidence intervals for sensitivity and specificity were calculated using the Clopper-Pearson method. Data analysis was conducted using IBM SPSS Statistics for Windows, Version 23 (Released 2015; IBM Corp., Armonk, New York, United States).

## Results

This cross-sectional study was conducted on 50 patients with BI-RADS 3 breast lesions. The patients' ages ranged from 15 to 70 years (mean 42±9 years). The highest incidence of breast lesions was observed in the 41-50 year age group, accounting for 38% (N=19) of the cases. No cases of malignancy were identified in patients aged 15-40 years. Malignancy was more prevalent in the 51-70 year age group and among nulliparous women. Most of the lesions were located in the upper outer quadrant (Table 1).

**Table 1:** Demographic characteristics of the BI-RADS 3 breast lesions in each patient BI-RADS: Breast imaging reporting and data system.

Variable	Categories	Frequency	Percentage	Benign	Malignant
Age group	15-40	22	44%	22	0
	41-50	19	38%	18	1
	51-70	9	18%	6	3
Parity	Multiparous	43	86%	42	1
	Nulliparous	5	10%	2	3
	Unmarried	2	4%	2	-
Quadrant	Upper outer	21	42%	18	3
	Upper inner	10	20%	10	0
	Lower outer	9	18%	9	0
	Lower inner	10	20%	9	1

**Figure 2:**
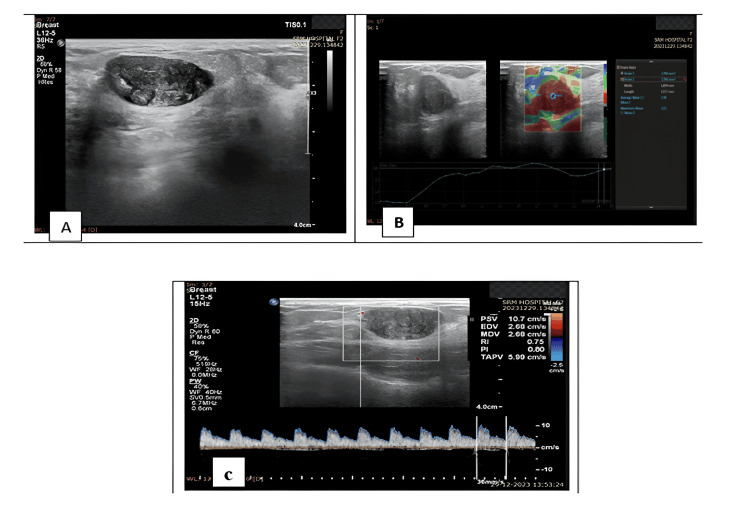
A 28-year-old female patient presented with a right breast lump A: Greyscale ultrasound showed a well-defined, wider-than-taller, oval-shaped hypoechoic lesion in the right upper outer quadrant. B: Strain elastography measured a strain ratio of 2.48. C: Doppler showed a PI of 0.8 and a RI of 0.75. FNAC confirmed the lesion as a fibroadenoma. PI: pulsatility index; RI: resistance index; FNAC: fine needle aspiration cytology.

Of the 50 lesions, 29 were located in the right breast and 21 in the left breast and 76% (N=38) showed no evidence of axillary lymph nodes, while 24% (N=12) presented with enlarged axillary lymph nodes with maintained fatty hilum. Calcifications were present in 10 cases, while 40 cases showed no evidence of calcification. The most common benign lesion identified on histopathology was fibroadenoma, which accounted for 84% (N=42) of cases (Table 2) (Figures 2A-2C).

**Table 2:** Comprehensive analysis of the characteristics of the breast lesions (frequency and percentage distribution) HPE: Histopathological examination

Variable	Categories	Frequency	Percentage
Lateralisation of lesions	Right breast	29	58%
	Left breast	21	42%
Axillary nodes	No	38	76%
	Yes	12	24%
Calcification	No	40	80%
	Yes	10	20%
Characterization of lesions based on HPE	Fibroadenoma	42	84%
	Giant fibroadenoma	2	4%
	Breast hamartoma	1	2%
	Benign phyllodes	1	2%
	Infiltrating ductal carcinoma	2	4%
	Mucinous carcinoma	1	2%
	Ductal carcinoma in situ	1	2%

**Figure 3:**
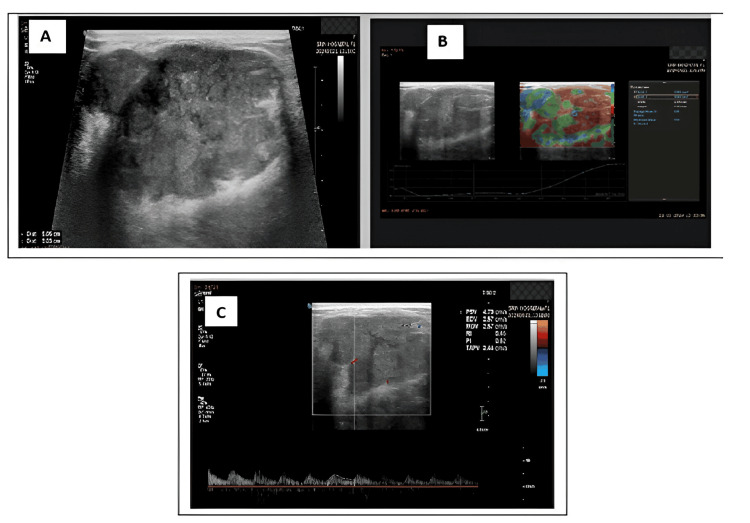
A 43-year-old female patient presented with right breast pain A: Greyscale ultrasound revealed a well-defined, wider-than-taller, macro-lobulated lesion with lobulated margins in the right upper outer quadrant and classified it as BI-RADS 3. B: Strain elastography showed a strain ratio of 2.29. C: Doppler revealed a PI of 0.62 and a RI of 0.45. However, FNAC confirmed the lesion as mucinous carcinoma. PI: pulsatility index; RI: resistance index; FNAC: fine needle aspiration cytology.

Among the four malignant lesions, two cases were identified as infiltrating ductal carcinoma (4%, N=2), one case as mucinous carcinoma (2%, N=1) (Figures 3A-3C), and one as ductal carcinoma in situ (2%, N=1).

Cut-off values for predicting malignancy in the BI-RADS 3 lesions were set at PI>1, RI>0.8, and strain ratio>3. Based on the above criteria, elastography strain ratio classified 92% (N=46) of cases as benign and 8% (N=4) as malignant. There was one false positive case and one false negative case. The elastography results demonstrated 75% sensitivity, 97.83% specificity, 75% positive predictive value (PPV), 97.83% negative predictive value (NPV), and a diagnostic accuracy of 96%. Doppler PI classified 90% (N=45) of patients as benign and 10% (N=5) as malignant. It had one false negative case and two false positive cases. The sensitivity was 75%, specificity was 95.65%, PPV was 60%, NPV was 97.78%, and the diagnostic accuracy was 94%. Doppler RI classified 90% (N=45) of patients as benign and 10% (N=5) as malignant. It had one false negative case and two false positive cases. The sensitivity was 75%, specificity was 95.65%, PPV was 60%, NPV was 97.78%, and the diagnostic accuracy was 94% (Table 3).

**Table 3:** Diagnostic performance of strain ratio and Doppler indices in differentiating benign and malignant breast lesions HPE: Histopathological examination; PI: pulsatility index; RI: resistance index.

Variables	Values	Frequency	HPE		Sensitivity	Specificity	Positive predictive value	Negative predictive value	Diagnostic accuracy
			Benign	Malignant					
Strain ratio	<3	46	45	1	75%	97.83%	75%	97.83%	96%
	>3	4	1	3					
Doppler PI	<1	45	44	1	75%	95.65%	60%	97.78%	94%
	>1	5	2	3					
Doppler RI	<0.8	45	44	1	75%	95.65%	60%	97.78%	94%
	>0.8	5	2	3					

## Discussion

Malignant breast lesions are typically stiff and hard in consistency; thus, the strain elastography technique can be utilized for the evaluation of breast cancers. Since most breast cancers exhibit increased vascularity, the Doppler PI and RI techniques are also employed for their detection. This study aimed to determine the sensitivity, specificity, PPV, NPV, and diagnostic accuracy of strain elastography and Doppler PI and RI indices for BI-RADS 3 breast lesions. The subjects in this study ranged in age from 15 to 70 years, with a mean age of 42±9 years. This is comparable to the research conducted by Cabuk et al., where the mean age was reported as 48.6 years [11]. The present study revealed a low cancer incidence among women younger than 25 years, aligning with the findings of Ha et al. (2015) which showed a similar low incidence in women of that age group [12].

In the current study involving 50 patients, histopathology confirmed that the most common breast pathology identified was fibroadenoma, accounting for 84% of patients (N=42). Previous studies by Chen et al. and Pradhan et al. indicated that the most common breast pathology diagnosed on histopathology was fibroadenoma, consistent with our findings [13,14]. Elia et al. (2021) assessed the role of strain elastography in characterizing breast masses and reported a sensitivity of 89.7%, specificity of 72.7%, PPV of 85.2%, and NPV of 80% [15]. 

Reghunath et al. (2021) [10] evaluated BI-RADS 3 and 4 lesions by combining elastography and Doppler techniques. Their findings indicated that the elastography technique with a strain ratio >3 had a sensitivity of 100%, specificity of 76.4%, PPV of 89.1%, and NPV of 100%. Cantisani et al. (2020) conducted a prospective cross-sectional comparative analysis between ultrasound BI-RADS, strain elastography, and shear wave elastography. For strain elastography, they reported a sensitivity of 89.2%, specificity of 76.6%, PPV of 87.1%, and NPV of 80% [16]. 

Sinha et al. (2020) conducted a prospective investigation to assess the classification of ambiguous breast lesions in the BI-RADS lexicon using strain elastography. They reported a sensitivity of 90%, specificity of 93.24%, PPV of 87.8%, NPV of 94.4%, and 92% accuracy [17]. Zhao et al. (2018) evaluated the role of strain elastography in characterizing breast masses and reported a sensitivity of 86.9%, specificity of 86.6%, PPV of 78.8%, NPV of 92%, and diagnostic accuracy of 82.6% [18]. Elkharbotly et al. (2015) indicated that strain elastography could serve as a sole diagnostic test, documenting a sensitivity of 83.35%, specificity of 88.1%, PPV of 75%, NPV of 92.5%, and diagnostic accuracy of 86.7% [19]. In our study, the technique's sensitivity for predicting malignancy was 75%, specificity was 97.83%, PPV was 75%, NPV was 97.83%, and diagnostic accuracy was 96% using a strain ratio>3 (Table 4).

**Table 4:** Comparison of the sensitivity, specificity, PPV, NPV, and diagnostic accuracy of strain elastography in our study with previous literature PPV: positive predictive value; NPV: negative predictive value

Author	Sensitivity	Specificity	Positive predictive value	Negative predictive value	Diagnostic accuracy
Elia et al. 2021 [15]	89.7%	72.7%	85.2%	80%	Not available
Reghunath et al. 2021 [10]	100%	76.4%	89.1%	100%	Not available
Cantisani et al. 2021 [16]	89.2%	76.6%	87.1%	80%	Not available
Sinha et al. 2020 [17]	90%	93.2%	87.8%	94.4%	92%
Zhao et al. 2018 [18]	86.9 %	86.6%	78.8%	92%	82.6%
Elkharbotly et al. 2015 [19]	83.35%	88.1%	75%	92.5%	86.7%
Our study (2024)	75%	97.83%	75%	97.83%	96.0%

Jain et al. (2022) reported that, in assessing breast mass lesions, Doppler PI yielded a sensitivity of 89%, specificity of 87.1%, PPV of 87.6%, NPV of 88.5%, and accuracy of 88.1%. For Doppler RI, they reported a sensitivity of 89%, specificity of 87.1%, PPV of 87.6%, and NPV of 88.5% [20]. 

Reghunath et al. reported that with a PI cut-off value of >1, the sensitivity was 81.8%, specificity was 76.4%, PPV was 87.1%, and NPV was 68.4%. For an RI cut-off value of >0.8, the sensitivity was reported as 81.8%, specificity as 82.3%, PPV as 90%, and NPV as 70% [10]. Keshavarz et al. (2018) reported 90% sensitivity and 85% specificity for Doppler PI and 85% sensitivity and 74% specificity for Doppler RI [21].

Sirous et al. reported a sensitivity of 70%, specificity of 98%, PPV of 93%, NPV of 88%, and diagnostic accuracy of 89% for Doppler PI. For Doppler RI, they reported a sensitivity of 75%, specificity of 97%, PPV of 90%, NPV of 89%, and diagnostic accuracy of 90% [9]. Elkharbotly et al. (2015) reported that, for Doppler RI, the sensitivity was 77.8%, specificity was 78.6%, PPV was 60.9%, NPV was 89.2%, and diagnostic accuracy was 86.7% [19]. In our study, the Doppler PI sensitivity was 75%, specificity was 95.65%, PPV was 60%, NPV was 97.78%, and diagnostic accuracy was 94%. Similarly, for Doppler RI, the sensitivity was 75%, specificity was 95.65%, PPV was 60%, NPV was 97.78%, and diagnostic accuracy was 94% (Table 5).

**Table 5:** Comparison of the sensitivity and specificity, PPV, NPV, and diagnostic accuracy of Doppler PI and RI in our study with previous literature PPV: positive predictive value; NPV: negative predictive value; PI: pulsatility index; RI: resistance index.

Author	Doppler indices	Sensitivity	Specificity	Positive predictive value	Negative predictive value	Diagnostic accuracy
Jain et al. 2022 [20]	PI	89%	87.1%	87.6%	88.5%	88.1%
	RI	89%	87.1%	87.6%	88.5%	88.1%
Reghunath et al. 2021 [10]	PI	81.8%	76.4%	93%	68.4%	Not available
	RI	81.8%	82.3%	87.1%	70%	Not available
Keshavarz et al. 2018 [21]	PI	90%	85%	Not available	Not available	Not available
	RI	85%	74%	Not available	Not available	Not available
Sirous et al. 2015 [9]	PI	70%	98%	93%	88%	89%
	RI	75%	97%	90%	89%	90%
Elkharbotly et al. 2015 [19]	RI	77.8%	78.6%	90%	89.2%	86.7%
Our study 2024	PI	75%	95.65%	60%	97.8%	94%
	RI	75%	95.65%	60%	97.8%	94%

The differential diagnosis in this study that appeared as a BI-RADS 3 lesion was mucinous carcinoma. On B-mode ultrasound, it presented as a well-defined, wider-than-tall, macrolobulated lesion with lobulated margins. On strain elastography, it showed a strain ratio of 2.29. Doppler revealed a PI of 0.62 and an RI of 0.45. However, FNAC confirmed the lesion as mucinous carcinoma (Figure 3).

This study had some limitations. Since tissue compression influences the strain ratio, adequate pressure is essential during examination. If excessive pressure is applied, incorrect diagnoses can occur. Due to its soft composition, false negatives may arise in cases of mucinous carcinoma. Additionally, false positives can occur in cases of highly increased cellular masses, such as giant fibroadenomas. Furthermore, the sample size in our study was relatively small, resulting in limited follow-up and evaluation of cases.

## Conclusions

In the evaluation of breast masses, B-mode ultrasonography serves as the first-line imaging modality and is especially effective in the initial assessment and categorization according to the BI-RADS system. A conservative approach is often taken for BI-RADS 3 lesions, considered probably benign or with a low likelihood of malignancy, and typically involves periodic follow-up imaging. However, incorporating additional diagnostic tools, such as strain elastography and Doppler PI and RI, can significantly enhance the accuracy of differentiating benign from malignant lesions. These supplementary modalities offer valuable information on tissue characteristics and vascularity, aiding in more precise assessment, and importantly, reducing the number of unnecessary biopsies.

Among these adjunct tools, strain elastography has shown particular promise, demonstrating higher diagnostic accuracy than Doppler PI and RI. This method evaluates tissue stiffness, a key indicator of potential malignancy, with greater reliability. The use of strain elastography along with the Doppler indices can support clinicians in making more informed decisions. These methods can also minimize invasive procedures and the associated patient anxiety while ensuring close monitoring of potentially suspicious findings. By refining the management of BI-RADS 3 lesions, this multimodal approach offers a balanced strategy that prioritizes patient safety and reduces healthcare costs.
